# Hearing loss and executive functions – results from a population-based cohort study

**DOI:** 10.1371/journal.pone.0351409

**Published:** 2026-06-22

**Authors:** Julia Döge, Daniëlle Otten, Berit Hackenberg, Karoline O’Brien, Manfred E. Beutel, Isabel Heinrich, Jörn M. Schattenberg, Stavros V. Konstantinides, Thomas Münzel, Karl J. Lackner, Irene Schmidtmann, Julian Chalabi, Alexander K. Schuster, Philipp S. Wild, Christoph Matthias, Katharina Bahr-Hamm

**Affiliations:** 1 Department of Otorhinolaryngology, University Medical Center of the Johannes Gutenberg-University Mainz, Germany; 2 Department of Psychosomatic Medicine and Psychotherapy, University Medical Center of the Johannes Gutenberg-University Mainz, Germany; 3 Department of Psychiatry and Psychotherapy, University Medical Center of the Johannes Gutenberg-University Mainz, Germany; 4 Center for Thrombosis and Hemostasis, University Medical Center of the Johannes Gutenberg-University Mainz, Germany; 5 Department of Cardiology - Cardiology I, University Medical Center of the Johannes Gutenberg-University Mainz, Germany; 6 Institute for Clinical Chemistry and Laboratory Medicine, University Medical Center of the Johannes Gutenberg-University Mainz, Germany; 7 Institute of Medical Biostatistics, Epidemiology and Informatics, University Medical Center of the Johannes Gutenberg-University Mainz, Germany; 8 Preventive Cardiology and Preventive Medicine – Department of Cardiology, University Medical Center of the Johannes Gutenberg-University Mainz, Germany; 9 Department of Ophthalmology, University Medical Center of the Johannes Gutenberg-University Mainz, Germany; 10 DZHK (German Center for Cardiovascular Research), partner site RhineMain, Mainz, Germany; 11 Department of Internal Medicine II, Saarland University Medical Center, Homburg, Germany; 12 Saarland University, Saarbruecken, Germany; Shiraz University of Medical Sciences, IRAN, ISLAMIC REPUBLIC OF

## Abstract

**Introduction:**

Hearing loss is linked to cognitive decline, yet its relation to specific executive functions remains unclear. This study examines associations between planning ability, pure-tone audiometry and speech-in-noise performance.

**Methods:**

Data were drawn from the Gutenberg Health Study (GHS), a large population-based cohort at the University Medical Center Mainz. Participants with complete pure-tone audiometry and planning ability data were included (*N* = 4,458; 2,314 males (51.9%), 2,144 (48.1%) females; mean age 58.0 ± 10.1 years, range 40–80). Audiologic testing included air- and bone-conduction pure-tone audiometry; hearing impairment was defined by WHO threshold. Speech-in-noise perception was measured using the German Matrix Test “Oldenburg Satztest”. Planning ability was assessed using the Freiburg version of the Tower of London. Associations were analyzed with linear and logistic regression.

**Results:**

Hearing loss (HL ≥ 20 dB; both ears) revealed a significant negative association with planning ability total score (β = −0.346, p < 0.01), but not significantly with clinical executive dysfunction (<16. Percentile total TOL) (OR=1.109, p = 0.43). Higher planning ability reduced the likelihood of hearing impairment (OR=0.97, p = 0.006) and was linked to better speech-in-noise perception (lower OLSA SRT, β = −0.035, p = 0.001).

**Conclusion:**

Hearing loss is linked to reduced planning ability, while higher planning ability decreases the likelihood of hearing impairment and increased speech perception in noise. These findings support models proposing that hearing loss imposes additional demands on working memory and attention. Longitudinal studies are needed to clarify causal directions and the potential role of interventions.

## Introduction

Hearing loss is a global health issue with a significant impact on quality of life. Approximately 1.5 billion people worldwide are affected by hearing loss, and this number is expected to rise continuously [1]. The prevalence of hearing impairment increases with age, yet many individuals with hearing loss are underserved [2]. Hearing loss can affect various aspects of an individual’s life, including cognitive decline and an increased risk of dementia [3,4]. The use of hearing aids has been linked to better cognitive function and a slower decline [5]. Studies suggest that individuals with mild cognitive impairment experience greater difficulties in communicating, particularly in noisy situations [6]. Moreover, reduced executive functioning appears to be associated with central auditory processing deficits [7].

Despite these findings, representative and up-to-date data on the prevalence of hearing loss, speech perception in noise, and its correlation with planning ability remain limited.

Hearing loss and cognitive decline are strongly associated [8–10] and may serve as an early indicator of prodromal stages of cognitive impairment [11]. Peripheral hearing loss has been linked to poorer performance on global cognitive assessments [12] and deficits in specific cognitive domains, including verbal memory performance [13], executive function [12,14] and processing speed [15]. The risk of cognitive decline increases progressively with the severity of hearing loss [12].

Executive functions (EF) are high-level cognitive processes that allow individuals to regulate goal-directed behavior and adapt flexibly to changing circumstances. The core components include planning ability, initiation, cognitive flexibility, and monitoring [16]. These abilities are essential for setting goals, planning and executing actions, and adjusting to new or unexpected situations [17].

EF shows age-related differences [18] with a decline in older age [19]. A commonly used task to assess planning ability and aspects of problem solving is the Tower of London (TOL) [20]. The computerized TOL-Freiburg version (TOL-F) was developed in 2012 to improve the task´s psychometric properties for diagnostic applications and is a suitable tool for research and clinical applications [21,22].

Unterrainer et al. investigated age-related changes in TOL performance. Their findings demonstrated that genetic factors differentially predicted cognitive performance in older versus middle-aged adults. Furthermore, among older individuals, high levels of alcohol consumption, the use of antidepressant medication, and living without a partner emerged as significant negative predictors of cognitive functioning [23].

The impact and interrelationships of executive function components, particularly planning ability, concerning hearing loss have not yet been sufficiently explored. This study aims to provide further insight into these associations.

This research contributes to the existing literature by incorporating both pure-tone audiometry and speech-in-noise testing to assess hearing impairment concerning planning ability. Additionally, it examines the prevalence of hearing loss, speech-in-noise performance, hearing aid provision, and planning ability within a large population-based cohort.

## Materials and methods

### Design

The Gutenberg Health Study (GHS) is a large population-based cohort study. It was initiated in 2007 at the University Medical Center in Germany, and the first participants were recruited in April 2007. Participants were randomly selected from the residents´ registration office, ensuring that the study population is representative of the inhabitants of Mainz and the district of Mainz-Bingen. The study was approved by the local institutional review board (Ethics Commission of the State Chamber of Physicians of Rhineland-Palatine, reference no. 837.020.07). It was conducted in full compliance with the Declaration of Helsinki. Written informed consent was obtained from all subjects before participation in the study. At the 10-year follow-up, comprehensive otological assessments were added to the broad, interdisciplinary examination protocol. A detailed description of the study design can be found elsewhere [24].

### Sample description

For the 10-year follow up, 12,423 participants took part in the subsequent examination. Of these, 3,306 participants had missing data for the TOL at both the 5- and 10-year follow-ups and were therefore excluded from the analyses. Due to missing data on pure-tone audiometry, 4,659 participants were additionally excluded. This resulted in a total sample of *N* = 4,458. This sample consisted of 2,314 males (51.9%) and 2,144 females (48.1%). The participants’ ages ranged from 40 to 80 years. The mean age was 58.0 (SD = 10.1) years, with females averaging slightly younger than males.

### Measurements

Participants underwent audiologic testing using pure-tone audiometry for air- and bone-conduction [25]. Hearing impairment was then calculated based on the revised WHO classification of hearing impairment (hearing threshold in the better hearing ear in decibels (dB), average across 0.5/1/2/4 kHz) (1).

The German Matrix Test “Oldenburg Satztest (OLSA)” was used to evaluate speech perception in noise [26,27]. The OLSA comprises sentences structured with five words and participants were asked to listen to the sentences and select the perceived words out of a matrix. The test uses a randomized adaptive procedure in which the speech level was fixed at 65 dB SPL, while the noise level was adaptively adjusted based on the number of correctly identified words. In the S0N0 condition, both speech and noise were presented from the front (0° azimuth).

The test was conducted in two consecutive phases: a trial run and a test run, each consisting of 20 sentences. The speech reception threshold (SRT) was recorded for each participant across both phases.

To assess planning ability and aspects of problem-solving, the computerised TOL-Freiburg version [21] was used, and participants’ ages ranged between 40 and 80 years. This version of the TOL was implemented in the Vienna Test System (https://marketplace.schuhfried.com/de/tol). It features three rods of varying heights and three colored balls (red, yellow, and blue) to replace the original green ball, making it suitable for individuals with red-green color blindness. Participants must transform a start state into a goal state in the minimum number of moves, following specific rules, while the program records any rule violations. Beyond the planning demands associated with the minimum number of moves, a TOL-F problem set that can be solved in four, five, or six moves is also considered (eight problem items each, resulting in an overall planning accuracy of 24 problems at maximum). The number of correctly solved tasks (range 0–24) was recorded. Scores at or below the 16th percentile rank, age dependent, were considered clinically significant. A detailed description of the study design can be found elsewhere [22,28].

Socioeconomic status (SES) was calculated according to Lampert and Kroll [29]. It has a sum value of 3 (lowest) to 21 (highest) and is formed from the characteristics’ highest educational attainment, household income and position in the occupation.

### Analyses

Continuous variables are presented as mean (standard deviation, SD) and tested with T-test, or if |skewness| > 1 as median (Q1, Q3) and tested with U-Test. Binary variables are described by relative and absolute frequencies and tested by chi-square test. All analyses were exploratory, with p-values (*P*) considered as a continuous measure of statistical evidence.

Logistic regression models were used to examine the likelihood of a clinically relevant TOL outcome as a function of hearing loss, with the TOL outcome as the dependent variable and hearing impairment as the independent variable. Additionally, logistic regression was applied to assess the probability of hearing impairment based on the TOL score, treating hearing impairment as the dependent variable and TOL total score as the predictor. Linear regression models were conducted to evaluate hearing impairment scores and OLSA values (dependent variables) concerning the TOL total score (independent variable). Furthermore, linear regression models examined the TOL total score (dependent variable) as a function of hearing impairment (independent variable). All statistical analyses were completed in R version 4.2.1 (2022-06-23 ucrt): R Core Team (2022).

## Results

### Descriptives

The overall prevalence of hearing loss across all levels of impairment was 43.0% (1918/4458). Hearing impairment was categorized into the following degrees: 30.5% of the cohort exhibited mild impairment, 9.8% moderate impairment, 2.2% moderately severe impairment, 0.3% severe impairment, 0.0% profound impairment, and 0.1% showed complete impairment. See Table 1 for the full results. Hearing impairment increased significantly with age (p < 0.001) and the hearing ability of females was significantly better than that of males (p < 0.001).

**Table 1:** Demographics and prevalence of hearing loss and planning ability.

	All (n)	Men (n)	Women (n)	P Value
**n**	4,458	2,314	2,144	
**Average Age years (SD)**	58.0 (10.1)	58.4 (10.2)	57.6 (10.0)	0.009
**TOL, total**	14.40 (3.62)	14.93 (3.61)	13.84 (3.54)	<0.001
**Set of 4**	7.00 (6.00/8.00)	7.00 (7.00/8.00)	7.00 (6.00/8.00)	<0.001
**Set of 5**	4.50 (1.70)	4.68 (1.71)	4.32 (1.66)	<0.001
**Set of 6**	2.92 (1.88)	3.17 (1.92)	2.67 (1.81)	<0.001
**Cancelled courses**	9.9% (332/3367)	8.9% (154/1723)	10.8% (178/1644)	0.073
**<16. percentile of total TOL**	10.8% (363/3367)	8.9% (153/1723)	12.8% (210/1644)	<0.001
**Grades of hearing loss according to WHO**	All (n)	Men (n)	Women (n)	
**n**				
**Normal hearing**	57.0% (2540/4458)	52.9% (1224/2314)	61.4% (1316/2144)	
**Mild hearing loss**	30.5% (1361/4458)	33.0% (763/2314)	27.9% (598/2144)	
**Moderate hearing loss**	9.8% (438/4458)	10.9% (253/2314)	8.6% (185/2144)	
**Moderately severe hearing loss**	2.2% (98/4458)	2.7% (62/2314)	1.7% (36/2144)	
**Severe hearing loss**	0.3% (13/4458)	0.2% (5/2314)	0.4% (8/2144)	
**Profound hearing loss**	0.0% (2/4458)	0.1% (2/2314)	0% (0/2144)	
**Complete or total hearing loss**	0.1% (6/4458)	0.2% (5/2314)	0.0% (1/2144)	
**Hearing loss (HL** ≥**20 dBdB; both ears)**	43.0% (1918/4458)	47.1% (1090/2314)	38.6% (828/2144)	<0.001

Table 1. Demographic characteristics of the study sample and prevalence of hearing loss (Grades of hearing loss according to WHO) and planning ability (TOL).

The overall mean OLSA SRT was −4.9 dB (−6.00 to −3.60). The mean OLSA was −4.7 dB for males and −5.2 dB for females. The OLSA-SRT values increase with age (−6.0 dB in the age group 40–49 years to −3.3 dB in the age group 70–80 years).

8.4% had a hearing aid on the right ear and 8.7% had a hearing aid on the left ear.

Across the entire cohort, 10.8% of participants exhibited clinically significant TOL performance (scores below the 16th percentile). Females showed a significantly higher prevalence of clinically significant test results compared to males (p < 0.001). The mean TOL total score was 14.4 (SD = 3.62). TOL total scores decreased with age, from a mean of 15.71 in the 40–49 age group to 12.00 in the 70–80 age group (p < 0.001).

Descriptive results are displayed in Table 1.

### Regressions

The linear regression model revealed that hearing impairment (HL ≥ 20 dB; both ears) was significantly negatively associated with the TOL total score (β = −0.346, 95% CI −0.587 to −0.0105, p = 0.005). Furthermore, higher age, lower SES, and female sex were associated with reduced TOL scores. In the logistic regression model for executive dysfunction (< 16th Percentile total TOL), hearing loss (HL ≥ 20 dB; both ears) as a predictor did not reach statistical significance (OR=1.109, p = 0.43). When divided into categories according to WHO, hearing loss in categories did not reach statistical significance as a predictor (mild hearing loss: OR=1.139, p = 0.34; moderate hearing loss: OR=0.891, p = 0.65; moderately severe hearing loss: OR=1.649, p = 0.29; severe hearing loss: OR=2.752, p = 0.23).

**Fig 1:**
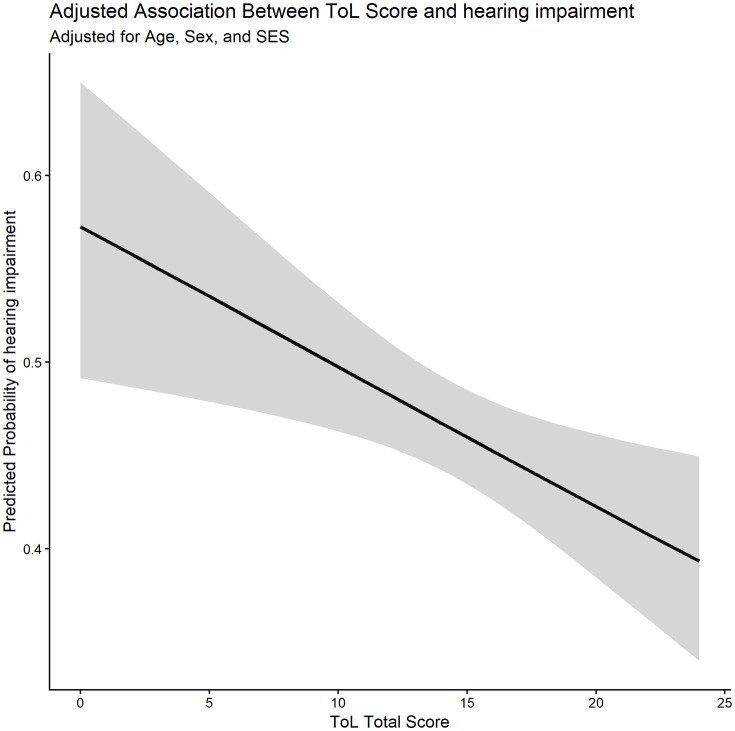
Predicted probability of hearing impairment by Tower of London total score (TOL). The probabilities were derived from a multivariable logistic regression model adjusted for age, sex, and socioeconomic status. The solid line represents estimated probabilities, and the shaded area indicates 95% confidence intervals.

Using executive function as a predictor and hearing impairment and speech perception in noise as outcome variables, the logistic regression model showed that a higher TOL total score significantly decreased the likelihood of hearing impairment (OR=0.97, 95% CI 0.949–0.991, p = 0.006). Fig 1 shows marginal predicted probabilities derived from the adjusted logistic regression model.

The linear regression model revealed that a higher TOL total score was significantly associated with a reduced overall mean of OLSA SRT (β = −0.035, 95% CI −0.035 to −0.014, p = 0.001).

**Fig 2:**
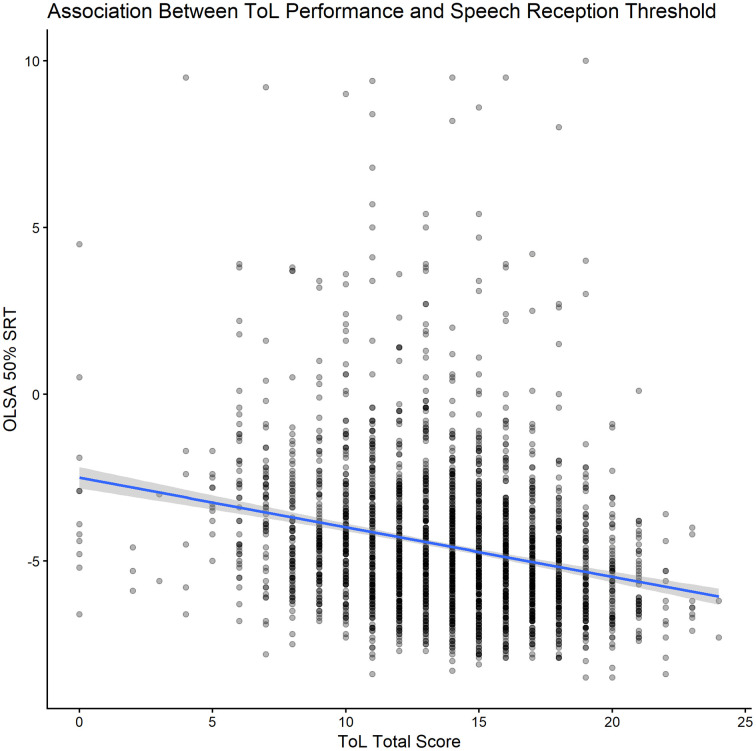
Association between Tower of London (TOL) total score and speech reception threshold in noise (OLSA 50% SRT). Points represent individual observations. The solid line indicates the fitted linear regression, and the shaded area represents the 95% confidence interval. Probabilities were derived from a linear regression model adjusted for age, sex, and socioeconomic status.

Fig 2 illustrates the negative association between TOL performance and OLSA 50% SRT.

Regression results are displayed in Tables 2,3 and 4.

**Table 2:** Association between TOL total score and hearing impairment.

TOL total Score	Beta [95% CI]	p-value
Hearing Impairment	−0.346[−0.587; −0.105]	0.005*
Sex (Women)	−1.027[−1.228; −0.825]	<0.001*
Age	−0.110[−0.121; −0.098]	<0.001*
SES	0.132[0.108; 0.156]	<0.001*

OR= Odds ratio, CI = confidence interval, * represents p-values with statistical significance.

Table 2. Linear regression model presenting odds ratios (OR) to examine the association between the TOL total Score (dependent variable) and the presence of hearing Impairment (HL ≥ 20 dB; both ears; independent variable), adjusted for sex and age.

**Table 3:** Association between hearing impairment and TOL total score.

Hearing Impairment	OR [95% CI]	p-value
TOL total Score	0.970 [0.949; 0.991]	0.006*
Sex (Women)	0.573 [0.493; 0.666]	<0.001*
Age	1.141 [1.131; 1.152]	<0.001*
SES	0.928 [0.912; 0.944]	<0.001*

OR= Odds ratio, CI = confidence interval, * represents p-values with statistical significance.

Table 3. Logistic regression model presenting odds ratios (OR) to examine the association between the presence of hearing Impairment (HL ≥ 20 dB; both ears; dependent variable) and TOL total Score (independent variable), adjusted for sex and age.

**Table 4:** Association between OLSA and TOL total score.

OLSA SRT	Beta [95% CI]	p-value
TOL total Score	−0.035 [−0.056; −0.014]	0.001*
Sex (Women)	−0.577 [−0.724; −0.431]	<0.001*
Age	0.097 [0.089; 0.105]	<0.001*
SES	−0.091 [−0.109; −0.074]	<0.001*

OR= Odds ratio, CI = confidence interval, * represents p-values with statistical significance.

Table 4. Linear regression model presenting odds ratios (OR) to examine the association between the presence of OLSA SRT (dependent variable) and TOL total Score (independent variable), adjusted for sex and age.

OR= Odds ratio, CI = confidence interval, * represents p-values with statistical significance.

## Discussion

The present study provides insights into the relationship between hearing loss, specifically pure-tone audiometric thresholds, and speech-in-noise perception, and planning ability.

Our findings indicate that hearing impairment influences executive function and vice versa. Hearing loss significantly reduced planning ability and at the same time a higher executive function level (i.e., planning ability) decreased the likelihood of hearing impairment. Furthermore, better planning ability increased speech perception in noise.

Moreover, higher age, lower SES, and female sex were linked to poorer TOL performance, indicating a potential impact of demographic and socioeconomic factors on cognitive planning. Consistent with these findings, Unterrainer et al. analyzed longitudinal data from the GHS cohort to investigate age-related changes in TOL performance and found, among others, living alone to be a significant adverse factors affecting cognitive functioning in the older subgroup [23].

Both planning ability and speech perception in noise rely on cognitive functions such as attention, working memory, and cognitive flexibility. Difficulty in understanding speech in noise may indicate impairments in these underlying executive functions, potentially leading to deficits in planning ability as well. Furthermore, both functions are linked to (pre-)frontal brain regions [20,30,31], suggesting that impairments in these areas could affect both functions.

The logistic regression model showed that better planning ability (as reflected by higher TOL scores) was associated with a decreased likelihood of hearing impairment. This suggests that individuals with better cognitive functions are less likely to experience hearing loss due to better auditory processing abilities.

Our findings align with theoretical models suggesting that hearing loss imposes additional cognitive demands, particularly on working memory and attention. Peelle et al. posit that increased listening effort due to auditory degradation depletes cognitive resources, which could in turn impact executive functions such as planning ability [30]. This is consistent with our results, which indicate that poorer planning performance is associated with hearing loss.

Studies indicate that age-related hearing loss is a possible biomarker and risk factor for later cognitive decline, cognitive impairment and dementia [32]. However, current evidence remains insufficient to conclusively determine the role of social isolation as a mediator in this relationship. Given that planning ability—a key executive function—is sensitive to cognitive decline, it is plausible that social isolation, which often arises as a consequence of hearing loss, could contribute to impairments in planning through reduced social engagement and cognitive stimulation. Dhanda et al. showed, however, that while hearing threshold levels clearly affect later cognitive outcomes and dementia diagnosis, there is still not enough evidence to clarify the mediating role of social isolation [33]. Within the framework of the GHS, Döge et al. demonstrated that hearing loss is associated with loneliness [34], while Unterrainer et al. found that living without a partner negatively influences planning performance [23]. These findings further support the notion that social and living conditions may play an important role in the relationship between hearing loss and planning ability.

A large proportion of participants (43%) had hearing loss, with the majority experiencing mild hearing loss (30.5%). Mild hearing loss typically causes only minor impairments in speech comprehension, both in quiet and noisy environments, whereas more severe hearing loss leads to greater difficulties. This could explain why individuals with hearing loss revealed lower executive function, as indicated by the TOL total score; however, no significant effect on clinically significant executive dysfunction was found. Mild hearing loss may not create a sufficient cognitive burden to impact planning ability systematically.

Previous research has linked hearing loss to reduced cognitive function and an increased risk of dementia [9,35,36]. Hearing loss is not only associated with general cognitive decline but can also impair specific executive functions [14]. Since planning ability depends strongly on cognitive processes such as working memory, cognitive flexibility, and attention, hearing loss may also hurt this function. These findings support our results, showing that hearing loss is linked to poorer total TOL performance and underline the importance of detection and treatment of hearing loss in order to prevent impairment of planning abilities.

### Strengths and limitations

Our study has several strengths, including a large, population-based cohort with extensive health data, standardized protocols for hearing assessment—incorporating both standardized pure-tone audiometry and speech-in-noise testing—and a systematic evaluation of planning ability. Importantly, the study included a broad age range (40–80 years) rather than focusing solely on older adults. However, some limitations must be acknowledged. The cross-sectional design of the hearing loss and planning ability data precludes causal inferences. Moreover, longitudinal data on hearing loss are currently lacking, limiting further analyses in this regard. Potential confounders, such as cognitive reserve, and overall health status, could have influenced the observed associations between hearing loss and planning ability. Additionally, the number of participants using hearing aids or experiencing severe to profound hearing loss is relatively small, and no longitudinal assessment has been conducted to evaluate changes in TOL outcomes following hearing aid fitting.

## Conclusion

This study highlights a significant bidirectional relation between hearing loss and planning ability. Hearing impairment was linked to reduced executive functioning, as measured by the TOL total score, while stronger planning ability was associated with better hearing and improved speech perception in noise. These findings support models suggesting that hearing loss increases cognitive demands, affecting key functions like attention and working memory. Early detection and treatment of hearing loss may help preserve cognitive health. Longitudinal studies are needed to confirm causality and explore the potential benefits of interventions targeting both auditory and cognitive domains in aging populations.
