# Supplementary material for: Hearing loss and executive functions – results from a population-based cohort study
Source: PLoS One. 2026 Jun 22;21(6):e0351409. doi: 10.1371/journal.pone.0351409 (PMC13286164; doi:10.1371/journal.pone.0351409)
Supplement: Attachment — Submitted filename: Response_to_Reviewer.docx [file pone.0351409.s002.docx]

**Response to the Editor and Reviewer**

We thank the Editor and the Reviewer for the careful evaluation of our manuscript and for the constructive comments and suggestions. We appreciate the opportunity to improve our work.

We have revised the manuscript accordingly. In response to the journal requirements, formatting issues have been corrected. We have also addressed the reviewer’s suggestions, including the addition of the proposed figure and clarification of the methodological aspects as requested.

All changes are highlighted in the revised manuscript. In addition to a clean version, a marked-up version showing all revisions has been provided for ease of reference.

Below, we provide a detailed, point-by-point response to each comment.

We hope that these revisions fully address the editorial and reviewer comments. We are grateful for the opportunity to revise our manuscript and are happy to provide any further clarification if required.

Thank you for your consideration.

Best regards,

Julia Döge

**Journal Requirements**

**Requirement 1:** File Naming and Style Templates

**Response:** Thank you for this note. We have carefully checked the manuscript and all associated files, including figures, and updated them to fully comply with PLOS ONE’s style requirements and file naming conventions.

**Requirement 2:** Ethical Approval Document

**Response:** As requested, we have uploaded a copy of the original ethical approval document (837.020.07) from 2007 and a englisch translation.

**Requirement 3:** Funding Statement

**Response:** We have removed all funding-related text from the manuscript outside the Funding Statement section. The Funding Statement itself remains unchanged and reads as follows:

“The Gutenberg Health Study (GHS) is funded through the government of Rhineland‐Palatinate (“Stiftung Rheinland‐Pfalz für Innovation”, contract AZ 961‐386261/733), the research program “Wissen schafft Zukunft” and “Center for Translational Vascular Biology (CTVB)” of the Johannes Gutenberg University of Mainz, and its contracts with Boehringer Ingelheim, and PHILIPS Medical Systems, including an unrestricted grant for the Gutenberg Health Study. Philipp S Wild is funded by the Federal Ministry of Education and Research (BMBF 01EO1503). Philipp S Wild and Thomas Münzel are PI of the German Center for Vascular Research (DZHK Rhein Main).

The funders had no role in study design, data collection and analysis, decision to publish, or preparation of the manuscript.”

As requested, we have included the Funding Statement in the cover letter.  We kindly request that you update the online submission form accordingly.

**Requirement 4:** Data Availability Statement

**Response:** The study includes clinical data from a large, population-based cohort with ongoing follow-up examinations. In order to maintain scientific integrity and adhere to the approved ethics protocol, the data cannot be shared publicly outside of controlled access procedures.

Qualified researchers may request access to the data on-site at the local database. Requests are reviewed and coordinated by the Coordination Committee of the Gutenberg Health Study at the University Medical Center Mainz (Email: info@ghs-mainz.de). Access is granted based on a written research proposal and evaluated by the committee, which includes representatives from all participating departments.

During revision, we identified a minor numerical error in the Abstract concerning the reported odds ratio and corresponding p-value. This has been corrected. The correction does not affect the results, interpretation, or conclusions of the study.

**Reviewer Comments**

**Reviewer comment 1:** Clarification of methodology
**Response:** We appreciate this comment and have clarified the methodological description in the Methods section (page 8, lines 209–212):

*„The test uses a randomized adaptive procedure in which the speech level was fixed at 65 dB SPL, while the noise level was adaptively adjusted based on the number of correctly identified words. In the S0N0 condition, both speech and noise were presented from the front (0° azimuth).“*

**Reviewer comment 2:** Suggestion to include a graphical illustration
**Response:** Thank you for this helpful suggestion. We have added two new figures to illustrate our findings.

Figure 1 shows marginal predicted probabilities of hearing impairment by Tower of London total score, derived from the adjusted logistic regression model (page 12, lines 299-304). Figure 2 illustrates the association between Tower of London performance and OLSA 50% SRT (page 13, lines 308-313).
