# Supplementary material for: Hearing loss and executive functions – results from a population-based cohort study
Source: PLoS One. 2026 Jun 22;21(6):e0351409. doi: 10.1371/journal.pone.0351409 (PMC13286164; doi:10.1371/journal.pone.0351409)
Supplement: Peer review history

# Peer review: Decision Letter 0

**DOI:** 10.1371/journal.pone.0351409.r001

**Authors:** Ali Faramarzi

11 Feb 2026

PONE-D-25-59707

Hearing Loss and Executive Functions – results from a population-based cohort study

PLOS One

Dear Dr. Döge,

Thank you for submitting your manuscript to PLOS ONE. After careful consideration, we feel that it has merit but does not fully meet PLOS ONE’s publication criteria as it currently stands. Therefore, we invite you to submit a revised version of the manuscript that addresses the points raised during the review process.

Please submit your revised manuscript by Mar 28 2026 11:59PM. If you will need more time than this to complete your revisions, please reply to this message or contact the journal office at plosone@plos.org. When you're ready to submit your revision, log on to https://www.editorialmanager.com/pone/ and select the 'Submissions Needing Revision' folder to locate your manuscript file.

Please include the following items when submitting your revised manuscript:

- A letter that responds to each point raised by the academic editor and reviewer(s). You should upload this letter as a separate file labeled 'Response to Reviewers'.
- A marked-up copy of your manuscript that highlights changes made to the original version. You should upload this as a separate file labeled 'Revised Manuscript with Track Changes'.
- An unmarked version of your revised paper without tracked changes. You should upload this as a separate file labeled 'Manuscript'.

-->If you would like to make changes to your financial disclosure, please include your updated statement in your cover letter. Guidelines for resubmitting your figure files are available below the reviewer comments at the end of this letter.

If applicable, we recommend that you deposit your laboratory protocols in protocols.io to enhance the reproducibility of your results. Protocols.io assigns your protocol its own identifier (DOI) so that it can be cited independently in the future. For instructions see: https://journals.plos.org/plosone/s/submission-guidelines#loc-laboratory-protocols. Additionally, PLOS ONE offers an option for publishing peer-reviewed Lab Protocol articles, which describe protocols hosted on protocols.io. Read more information on sharing protocols at https://plos.org/protocols?utm_medium=editorial-email&utm_source=authorletters&utm_campaign=protocols.

We look forward to receiving your revised manuscript.

Kind regards,

Ali Faramarzi, MD, MPH

Academic Editor

PLOS One

Journal requirements:

When submitting your revision, we need you to address these additional requirements.

1. Please ensure that your manuscript meets PLOS ONE's style requirements, including those for file naming. The PLOS ONE style templates can be found at

https://journals.plos.org/plosone/s/file?id=wjVg/PLOSOne_formatting_sample_main_body.pdf and

https://journals.plos.org/plosone/s/file?id=ba62/PLOSOne_formatting_sample_title_authors_affiliations.pdf

2. As mentioned in our previous correspondence, please upload a copy of the original ethical approval document (837.020.07) from 2007. We require this ethical approval document since you have accessed identifying information about participants in the original study for the follow-up portion of the study described in this manuscript.

3. Thank you for stating the following in the Funding Section of your manuscript:

“The Gutenberg Health Study (GHS) is funded through the government of Rhineland‐Palatinate (“Stiftung Rheinland‐Pfalz für Innovation”, contract AZ 961‐386261/733), the research program “Wissen schafft Zukunft” and “Center for Translational Vascular Biology (CTVB)” of the Johannes Gutenberg University of Mainz, and its contracts with Boehringer Ingelheim, and PHILIPS Medical Systems, including an unrestricted grant for the Gutenberg Health Study. Philipp S Wild is funded by the Federal Ministry of Education and Research (BMBF 01EO1503). Philipp S Wild and Thomas Münzel are PI of the German Center for Vascular Research (DZHK Rhein Main).

The funders had no role in study design, data collection and analysis, decision to publish, or preparation of the manuscript.”

We note that you have provided funding information that is currently declared in your Funding Statement. However, funding information should not appear in the Acknowledgments section or other areas of your manuscript. We will only publish funding information present in the Funding Statement section of the online submission form.

Please remove any funding-related text from the manuscript and let us know how you would like to update your Funding Statement. Currently, your Funding Statement reads as follows:

“The Gutenberg Health Study (GHS) is funded through the government of Rhineland‐Palatinate (“Stiftung Rheinland‐Pfalz für Innovation”, contract AZ 961‐386261/733), the research program “Wissen schafft Zukunft” and “Center for Translational Vascular Biology (CTVB)” of the Johannes Gutenberg University of Mainz, and its contracts with Boehringer Ingelheim, and PHILIPS Medical Systems, including an unrestricted grant for the Gutenberg Health Study. Philipp S Wild is funded by the Federal Ministry of Education and Research (BMBF 01EO1503). Philipp S Wild and Thomas Münzel are PI of the German Center for Vascular Research (DZHK Rhein Main).

The funders had no role in study design, data collection and analysis, decision to publish, or preparation of the manuscript.”

Please include your amended statements within your cover letter; we will change the online submission form on your behalf.

4. We note that you have indicated that there are restrictions to data sharing for this study. For studies involving human research participant data or other sensitive data, we encourage authors to share de-identified or anonymized data. However, when data cannot be publicly shared for ethical reasons, we allow authors to make their data sets available upon request. For information on unacceptable data access restrictions, please see http://journals.plos.org/plosone/s/data-availability#loc-unacceptable-data-access-restrictions.

Before we proceed with your manuscript, please address the following prompts:

a) If there are ethical or legal restrictions on sharing a de-identified data set, please explain them in detail (e.g., data contain potentially identifying or sensitive patient information, data are owned by a third-party organization, etc.) and who has imposed them (e.g., a Research Ethics Committee or Institutional Review Board, etc.). Please also provide contact information for a data access committee, ethics committee, or other institutional body to which data requests may be sent.

b) If there are no restrictions, please upload the minimal anonymized data set necessary to replicate your study findings to a stable, public repository and provide us with the relevant URLs, DOIs, or accession numbers. Please see http://www.bmj.com/content/340/bmj.c181.long for guidelines on how to de-identify and prepare clinical data for publication. For a list of recommended repositories, please see https://journals.plos.org/plosone/s/recommended-repositories. You also have the option of uploading the data as Supporting Information files, but we would recommend depositing data directly to a data repository if possible.

Please update your Data Availability statement in the submission form accordingly.

If the reviewer comments include a recommendation to cite specific previously published works, please review and evaluate these publications to determine whether they are relevant and should be cited. There is no requirement to cite these works unless the editor has indicated otherwise.

Please review your reference list to ensure that it is complete and correct. If you have cited papers that have been retracted, please include the rationale for doing so in the manuscript text, or remove these references and replace them with relevant current references. Any changes to the reference list should be mentioned in the rebuttal letter that accompanies your revised manuscript. If you need to cite a retracted article, indicate the article’s retracted status in the References list and also include a citation and full reference for the retraction notice.

[Note: HTML markup is below. Please do not edit.]

Reviewers' comments:

Reviewer's Responses to Questions

**Comments to the Author**

1. Is the manuscript technically sound, and do the data support the conclusions?

The manuscript must describe a technically sound piece of scientific research with data that supports the conclusions. Experiments must have been conducted rigorously, with appropriate controls, replication, and sample sizes. The conclusions must be drawn appropriately based on the data presented.

Reviewer #1: Yes

**********

2. Has the statistical analysis been performed appropriately and rigorously?

Reviewer #1: Yes

**********

3. Have the authors made all data underlying the findings in their manuscript fully available?

The PLOS Data policy requires authors to make all data underlying the findings described in their manuscript fully available without restriction, with rare exception (please refer to the Data Availability Statement in the manuscript PDF file). The data should be provided as part of the manuscript or its supporting information, or deposited to a public repository. For example, in addition to summary statistics, the data points behind means, medians and variance measures should be available. If there are restrictions on publicly sharing data—e.g. participant privacy or use of data from a third party—those must be specified.

Reviewer #1: Yes

**********

4. Is the manuscript presented in an intelligible fashion and written in standard English?

PLOS ONE does not copyedit accepted manuscripts, so the language in submitted articles must be clear, correct, and unambiguous. Any typographical or grammatical errors should be corrected at revision, so please note any specific errors here.

Reviewer #1: Yes

**********

5. Review Comments to the Author

Please use the space provided to explain your answers to the questions above. You may also include additional comments for the author, including concerns about dual publication, research ethics, or publication ethics. (Please upload your review as an attachment if it exceeds 20,000 characters)

Reviewer #1: This manuscript presents a high-quality and well-conducted population-based study addressing the association between hearing loss, speech-in-noise perception, and executive functioning, specifically planning ability. The large sample size, the comprehensive audiological assessment, and the clear methodological approach substantially strengthen the scientific value and reliability of the findings. Overall, the study is highly relevant, methodologically sound, and clinically meaningful.

Major strengths:

1 The very large and representative cohort markedly increases the robustness and generalizability of the results.

2. The combined use of pure-tone audiometry and speech-in-noise testing (OLSA) provides a comprehensive evaluation of hearing function.

3. The clear statistical methodology and transparent reporting of regression analyses allow for good interpretability.

4. The focus on planning ability as a specific executive function adds valuable nuance beyond global cognitive screening.

Minor suggestions for improvement

1. Clarification of OLSA measurement setup

The description of the Oldenburg Sentence Test (OLSA) would benefit from more precise technical detail. Specifically, it would be helpful to explicitly report:

the measurement configuration (e.g. S0N0 condition), the noise presentation level (e.g. fixed noise at 65 dB SPL),

This additional information would improve reproducibility and methodological transparency, especially for audiological readers and future meta-analyses.

2. Visualization of regression relationships

The manuscript would benefit from the inclusion of a regression figure to visually illustrate the key associations, particularly:

TOL total score vs. hearing impairment (or PTA),

TOL total score vs. OLSA SRT.

A recommended option would be:

a scatter plot with regression line and confidence interval (e.g. TOL vs. OLSA SRT),

or a partial regression (adjusted) plot showing the association after controlling for age, sex, and SES,

alternatively, a marginal effects plot derived from the regression models.

This would significantly enhance interpretability and accessibility of the results, especially for interdisciplinary audiences, and improve the visual communication of effect directions and strengths.

Conclusion:

In summary, this manuscript represents a very strong contribution to the literature on hearing loss and cognition. With minor additions regarding methodological details of the OLSA and enhanced graphical presentation of regression findings, the manuscript would be further strengthened. I strongly support publication after these minor revisions.

**********

6. PLOS authors have the option to publish the peer review history of their article (what does this mean?). If published, this will include your full peer review and any attached files.

If you choose “no”, your identity will remain anonymous but your review may still be made public.

**Do you want your identity to be public for this peer review?**   For information about this choice, including consent withdrawal, please see our Privacy Policy.

Reviewer #1: **Yes:**  PD Dr. med. Susen Lailach

**********

[NOTE: If reviewer comments were submitted as an attachment file, they will be attached to this email and accessible via the submission site. Please log into your account, locate the manuscript record, and check for the action link "View Attachments". If this link does not appear, there are no attachment files.]

To ensure your figures meet our technical requirements, please review our figure guidelines: https://journals.plos.org/plosone/s/figures

You may also use PLOS’s free figure tool, NAAS, to help you prepare publication quality figures: https://journals.plos.org/plosone/s/figures#loc-tools-for-figure-preparation.

NAAS will assess whether your figures meet our technical requirements by comparing each figure against our figure specifications.

# Author comment: Author response to Decision Letter 1

**DOI:** 10.1371/journal.pone.0351409.r002

11 Mar 2026

Response to the Editor and Reviewer

We thank the Editor and the Reviewer for the careful evaluation of our manuscript and for the constructive comments and suggestions. We appreciate the opportunity to improve our work.

We have revised the manuscript accordingly. In response to the journal requirements, formatting issues have been corrected. We have also addressed the reviewer’s suggestions, including the addition of the proposed figure and clarification of the methodological aspects as requested.

All changes are highlighted in the revised manuscript. In addition to a clean version, a marked-up version showing all revisions has been provided for ease of reference.

Below, we provide a detailed, point-by-point response to each comment.

We hope that these revisions fully address the editorial and reviewer comments. We are grateful for the opportunity to revise our manuscript and are happy to provide any further clarification if required.

Thank you for your consideration.

Best regards,

Julia Döge

Journal Requirements

Requirement 1: File Naming and Style Templates

Response: Thank you for this note. We have carefully checked the manuscript and all associated files, including figures, and updated them to fully comply with PLOS ONE’s style requirements and file naming conventions.

Requirement 2: Ethical Approval Document

Response: As requested, we have uploaded a copy of the original ethical approval document (837.020.07) from 2007 and a englisch translation.

Requirement 3: Funding Statement

Response: We have removed all funding-related text from the manuscript outside the Funding Statement section. The Funding Statement itself remains unchanged and reads as follows:

“The Gutenberg Health Study (GHS) is funded through the government of Rhineland‐Palatinate (“Stiftung Rheinland‐Pfalz für Innovation”, contract AZ 961‐386261/733), the research program “Wissen schafft Zukunft” and “Center for Translational Vascular Biology (CTVB)” of the Johannes Gutenberg University of Mainz, and its contracts with Boehringer Ingelheim, and PHILIPS Medical Systems, including an unrestricted grant for the Gutenberg Health Study. Philipp S Wild is funded by the Federal Ministry of Education and Research (BMBF 01EO1503). Philipp S Wild and Thomas Münzel are PI of the German Center for Vascular Research (DZHK Rhein Main).

The funders had no role in study design, data collection and analysis, decision to publish, or preparation of the manuscript.”

As requested, we have included the Funding Statement in the cover letter. We kindly request that you update the online submission form accordingly.

Requirement 4: Data Availability Statement

Response: The study includes clinical data from a large, population-based cohort with ongoing follow-up examinations. In order to maintain scientific integrity and adhere to the approved ethics protocol, the data cannot be shared publicly outside of controlled access procedures.

Qualified researchers may request access to the data on-site at the local database. Requests are reviewed and coordinated by the Coordination Committee of the Gutenberg Health Study at the University Medical Center Mainz (Email: info@ghs-mainz.de). Access is granted based on a written research proposal and evaluated by the committee, which includes representatives from all participating departments.

During revision, we identified a minor numerical error in the Abstract concerning the reported odds ratio and corresponding p-value. This has been corrected. The correction does not affect the results, interpretation, or conclusions of the study.

Reviewer Comments

Reviewer comment 1: Clarification of methodology

Response: We appreciate this comment and have clarified the methodological description in the Methods section (page 8, lines 209–212):

„The test uses a randomized adaptive procedure in which the speech level was fixed at 65 dB SPL, while the noise level was adaptively adjusted based on the number of correctly identified words. In the S0N0 condition, both speech and noise were presented from the front (0° azimuth).“

Reviewer comment 2: Suggestion to include a graphical illustration

Response: Thank you for this helpful suggestion. We have added two new figures to illustrate our findings.

Figure 1 shows marginal predicted probabilities of hearing impairment by Tower of London total score, derived from the adjusted logistic regression model (page 12, lines 299-304). Figure 2 illustrates the association between Tower of London performance and OLSA 50% SRT (page 13, lines 308-313).

Attachment Submitted filename: Response_to_Reviewer.docx

# Peer review: Decision Letter 1

**DOI:** 10.1371/journal.pone.0351409.r003

**Authors:** Ali Faramarzi

27 May 2026

Hearing loss and executive functions – results from a population-based cohort study

PONE-D-25-59707R1

Dear Dr. Döge,

We’re pleased to inform you that your manuscript has been judged scientifically suitable for publication and will be formally accepted for publication once it meets all outstanding technical requirements.

Within one week, you’ll receive an e-mail detailing the required amendments. When these have been addressed, you’ll receive a formal acceptance letter and your manuscript will be scheduled for publication.

An invoice will be generated when your article is formally accepted. Please note, if your institution has a publishing partnership with PLOS and your article meets the relevant criteria, all or part of your publication costs will be covered. Please make sure your user information is up-to-date by logging into Editorial Manager at Editorial Manager® and clicking the ‘Update My Information' link at the top of the page. For questions related to billing, please contact billing support.

If your institution or institutions have a press office, please notify them about your upcoming paper to help maximize its impact. If they’ll be preparing press materials, please inform our press team as soon as possible -- no later than 48 hours after receiving the formal acceptance. Your manuscript will remain under strict press embargo until 2 pm Eastern Time on the date of publication. For more information, please contact onepress@plos.org.

Kind regards,

Ali Faramarzi, MD, MPH

Academic Editor

PLOS One

Additional Editor Comments (optional):

Reviewers' comments:

Reviewer's Responses to Questions

**Comments to the Author**

1. If the authors have adequately addressed your comments raised in a previous round of review and you feel that this manuscript is now acceptable for publication, you may indicate that here to bypass the “Comments to the Author” section, enter your conflict of interest statement in the “Confidential to Editor” section, and submit your "Accept" recommendation.

Reviewer #2: All comments have been addressed

**********

2. Is the manuscript technically sound, and do the data support the conclusions?

The manuscript must describe a technically sound piece of scientific research with data that supports the conclusions. Experiments must have been conducted rigorously, with appropriate controls, replication, and sample sizes. The conclusions must be drawn appropriately based on the data presented.

Reviewer #2: Yes

**********

3. Has the statistical analysis been performed appropriately and rigorously?

Reviewer #2: Yes

**********

4. Have the authors made all data underlying the findings in their manuscript fully available?

The PLOS Data policy requires authors to make all data underlying the findings described in their manuscript fully available without restriction, with rare exception (please refer to the Data Availability Statement in the manuscript PDF file). The data should be provided as part of the manuscript or its supporting information, or deposited to a public repository. For example, in addition to summary statistics, the data points behind means, medians and variance measures should be available. If there are restrictions on publicly sharing data—e.g. participant privacy or use of data from a third party—those must be specified.

Reviewer #2: Yes

**********

5. Is the manuscript presented in an intelligible fashion and written in standard English?

PLOS ONE does not copyedit accepted manuscripts, so the language in submitted articles must be clear, correct, and unambiguous. Any typographical or grammatical errors should be corrected at revision, so please note any specific errors here.

Reviewer #2: Yes

**********

6. Review Comments to the Author

Please use the space provided to explain your answers to the questions above. You may also include additional comments for the author, including concerns about dual publication, research ethics, or publication ethics. (Please upload your review as an attachment if it exceeds 20,000 characters)

Reviewer #2: The paper “Hearing loss and executive functions – results from a population-based cohort study” presents a well-structured and compelling contribution to the field. The research question is clearly articulated and addresses an interesting question. The authors demonstrate a solid understanding of the existing literature and position their work appropriately within it.

The paper is methodologically rigorous and well written. The results are presented clearly, with sufficient detail to support the claims made. The writing is concise and generally easy to follow, and the figures and tables effectively complement the text.

In summary, this is a high-quality paper that makes a meaningful contribution to the field. I recommend acceptance.

**********

7. PLOS authors have the option to publish the peer review history of their article (what does this mean?). If published, this will include your full peer review and any attached files.

If you choose “no”, your identity will remain anonymous but your review may still be made public.

**Do you want your identity to be public for this peer review?**   For information about this choice, including consent withdrawal, please see our Privacy Policy.

Reviewer #2: No

**********

# Editor report: Acceptance letter

**DOI:** 10.1371/journal.pone.0351409.r004

**Authors:** Ali Faramarzi

PONE-D-25-59707R1

PLOS One

Dear Dr. Döge,

I'm pleased to inform you that your manuscript has been deemed suitable for publication in PLOS One. Congratulations! Your manuscript is now being handed over to our production team.

At this stage, our production department will prepare your paper for publication. This includes ensuring the following:

* All references, tables, and figures are properly cited

* All relevant supporting information is included in the manuscript submission,

* There are no issues that prevent the paper from being properly typeset

You will receive further instructions from the production team, including instructions on how to review your proof when it is ready. Please keep in mind that we are working through a large volume of accepted articles, so please give us a few days to review your paper and let you know the next and final steps.

Lastly, if your institution or institutions have a press office, please let them know about your upcoming paper now to help maximize its impact. If they'll be preparing press materials, please inform our press team within the next 48 hours. Your manuscript will remain under strict press embargo until 2 pm Eastern Time on the date of publication. For more information, please contact onepress@plos.org.

You will receive an invoice from PLOS for your publication fee after your manuscript has reached the completed accept phase. If you receive an email requesting payment before acceptance or for any other service, this may be a phishing scheme. Learn how to identify phishing emails and protect your accounts at https://explore.plos.org/phishing.

If we can help with anything else, please email us at customercare@plos.org.

Thank you for submitting your work to PLOS ONE and supporting open access.

Kind regards,

PLOS ONE Editorial Office Staff

on behalf of

Dr. Ali Faramarzi

Academic Editor

PLOS One
